# Loving ACTion: An evaluation of an ACT‐based audio podcast intervention focussed on romantic and intimate relationships for adults with visible differences

**DOI:** 10.1111/bjhp.70096

**Published:** 2026-07-28

**Authors:** Maia Thornton, Emma Waite, Paul White, Anna Zarola, Yara Vizinho, Alex Clarke, Diana Harcourt

**Affiliations:** ^1^ Centre for Appearance Research, School of Social Sciences University of the West of England Bristol UK

**Keywords:** intervention, intimacy, psychosocial support, romantic relationships, sex, visible difference

## Abstract

**Objectives:**

Research suggests that some adults with visible differences may experience challenges related to romantic relationships, sex and physical intimacy. Loving ACTion is a self‐guided Acceptance and Commitment Therapy (ACT)‐based intervention delivered as an audio podcast series, co‐produced with adults with visible differences and designed as a supportive intervention for adults with any kind of visible difference seeking help to navigate challenges related to intimacy and romantic relationships. The present study aimed to evaluate the effectiveness of Loving ACTion using a mixed methods design.

**Methods:**

Seventy‐one adults with a range of visible differences completed an online mixed methods survey at three time points: (1) baseline, (2) post‐intervention and (3) 2‐month follow‐up. Changes in psychosocial outcomes were examined using paired *t*‐tests, and open‐ended responses were analysed using content analysis.

**Results:**

Between baseline and post‐intervention, there were significant improvements in appearance‐related distress in romantic relationships, body image life disengagement, fear of negative appearance evaluation and internal shame as well as significant improvements in body esteem, psychological flexibility and self‐compassion. These improvements were maintained at follow‐up, with additional improvements observed in valued living. Content analysis suggested that engaging with Loving ACTion positively impacted wellbeing and made a difference to participants' lives.

**Conclusions:**

These findings provide preliminary support for the potential value of this novel, accessible intervention for adults with visible differences navigating romantic and intimate relationships. Loving ACTion is now freely available at www.VisibleDifferenceSupportHub.com.

**Statement of Contribution**

**What is already known?**

- Visible differences can affect adults' experiences of intimacy and romantic relationships.
- Support for relationship and intimacy concerns in visible difference is limited.
- ACT shows promise for reducing appearance‐related distress in visible difference.

**What does this study add?**

- The first evaluation of an ACT‐based podcast for relationships and intimacy in visible difference.
- Loving ACTion improved relationship distress, self‐compassion and psychological flexibility.
- Findings support a novel, accessible support option for adults with visible differences.

## INTRODUCTION

A visible difference is defined as an appearance that differs from societal norms (Rumsey & Harcourt, 2004) and can be caused by a range of conditions (e.g., craniofacial conditions, skin conditions), acquired injuries (e.g., burn injuries, limb loss) or a result of medical treatments (e.g., surgical scarring). Although many individuals adjust well to their visible difference (Baillie et al., 2014; Egan et al., 2011), some adults can experience pervasive psychosocial challenges such as unwanted attention from others, social stigma and social avoidance (Rasset et al., 2022) as well as emotional challenges such as appearance concerns, negative self‐perceptions, low self‐esteem and fear of negative evaluation (Tiemens et al., 2013; van Dalen et al., 2020). One area of concern for people with a visible difference can be the development and maintenance of romantic and intimate relationships. Qualitative research has found that adults and adolescents with visible differences are aware of the value placed on physical appearance during dating and the development of romantic relationships (Sharratt et al., 2018; van Dalen et al., 2020). Some report feeling devalued in the context of dating and romantic relationships due to their appearance difference and feeling the need to compensate by either altering other aspects of their appearance (e.g., changing their body through exercise) or succeeding in other aspects of life (e.g., pursuing a good education or job) (Sharratt et al., 2018). Adults with a rare appearance‐affecting skin condition, ichthyosis, noted concerns about other peoples' negative reactions to their appearance difference, with some being worried that potential partners may be repulsed by their condition or that their relationship would not last (Mazereeuw‐Hautier et al., 2012). Furthermore, a survey with 181 adults with cleft lip and/or palate found that participants had lower satisfaction within their intimate relationships compared to the general population (Ardouin et al., 2021).

Adults with visible differences have also expressed concerns related to sex. For example, a qualitative study conducted in the United Kingdom with 22 adults with a range of visible differences found that participants reported reduced sexual desire, discomfort with being seen naked and rejecting advances from their partner or attempting to conceal their appearance difference during sexual activity (Sharratt et al., 2018). Negative impact on sexual desire and activity has also been found in individuals receiving treatment for burn injuries in Australia (Connell et al., 2015) as well as young people living in the United Kingdom with psoriasis, who have reported avoiding intimate situations and behaviours due to their skin condition (Fox et al., 2007).

Some adults with visible differences also describe condition‐specific concerns that may impact their experience of sex. For example, veterans with acquired visible differences (e.g., limb loss) have reported difficulties in learning new ways to engage in sex, for example, finding new sexual positions (Keeling & Sharratt, 2023). Such acquired visible differences can also lead to changes in roles within relationships (e.g., partners becoming caregivers), which can impact on the experience of intimacy (Lewis et al., 2024).

Disclosure and communication about a visible difference with a potential romantic partner has also been raised in the existing literature. A qualitative study with 15 adults with a range of conditions found some individuals with appearance differences that were less noticeable or easily concealed (e.g., by clothing) felt anxious about disclosing their visible difference (Sharratt et al., 2020). Some participants described a desire to avoid speaking about their visible difference, whereas others noted that it felt important to disclose to a new or potential partner before they had sex or were naked in front of someone for the first time (Sharratt et al., 2020). Many described the preparations they had made in advance of disclosure (e.g., planning when and how they would share the information). In addition, a study with 288 adults with a facial difference found that the type of disclosure influenced relationship outcomes (Bogart et al., 2023). Agentic disclosure (feeling obliged to disclose) was associated with lower relationship self‐concept (ability to develop intimacy and communicate with a partner) than those who reported engaging in autonomous disclosure (choosing to openly disclose). As such, disclosure in romantic relationships can be anxiety‐inducing to navigate for people with a visible difference and the circumstances of disclosure can impact relationship outcomes.

Research has also shown that some individuals report having a visible difference has had a positive influence in their relationships, such as feeling that their partner really appreciated them for who they are beyond their appearance and noting the value of emotional support, companionship and safety in their relationship (Sharratt et al., 2018). In an interview study of women with below‐knee prosthesis, participants reported that another person's response to their visible difference acted as a helpful assessment of their character and helped participants make judgements about their suitability as a partner (Mathias & Harcourt, 2014). Furthermore, in a mixed methods study exploring the experiences of adults with neurofibromatosis, many reported that their condition had no impact on their relationships (Bicudo et al., 2016). In summary, although research suggests that there may be some common challenges related to romantic relationships, it is important to consider that the impact of having a visible difference on romantic relationships can vary greatly and support needs cannot be generalized.

Although some psychosocial interventions for adults with visible differences are available (Muftin & Thompson, 2013; Norman & Moss, 2015; Zucchelli et al., 2022), these do not focus specifically on support needs related to romantic relationships and intimacy and they are mostly underpinned by Cognitive Behavioural Therapy (CBT), despite a limited evidence base for its effectiveness in this context (Zucchelli et al., 2018). Acceptance and Commitment Therapy (ACT) is a third‐wave CBT approach that is gaining an evidence base for use in supporting adults with a visible difference who experience difficult thoughts (e.g., people may stare) or feelings (e.g., anxiety about reactions of others), which may reflect their reality (Zucchelli et al., 2018). Unlike CBT, ACT does not frame these thoughts and feelings as maladaptive but focuses on reframing the individual's relationship to these internal experiences (Luoma et al., 2007). This encourages psychological flexibility, which is characterized by the ability to create space from difficult internal experiences, while promoting behaviour aligned with personal values.

Previous research has established that cognitive fusion and experiential avoidance (key components of psychological flexibility) partially mediate the relationship between body dissatisfaction and behavioural avoidance and appearance‐fixing behaviours in adults with a visible difference (Zucchelli et al., 2020). Several ACT‐based interventions have been found to be acceptable including an app (‘ACT It Out’) for adults with visible differences of any kind (Zucchelli et al., 2022) and an early intervention to support adults in adjusting to appearance changes following a burn injury (Shepherd et al., 2026). Additionally, ACT‐based psychoeducational self‐help guides have demonstrated favourable psychosocial changes among adults with facial palsy (Hotton et al., 2022). Existing research evidence therefore suggests that ACT‐based interventions could be an appropriate therapeutic approach for supporting adults with visible differences who have concerns related to intimacy and romantic relationships.

Loving ACTion is a self‐guided ACT‐based intervention co‐produced alongside adults with lived experience and delivered via a series of audio podcast episodes. The decision to create a podcast‐based intervention was informed by existing literature that suggests this can be an effective format for improving a variety of psychosocial outcomes, such as positive body image (Guest et al., 2019), self‐efficacy and psychological distress (Carrotte et al., 2025) and conveying health information (Semakula et al., 2017). Furthermore, an audio‐only podcast format can usefully lower barriers to help seeking for sensitive or potentially shame‐laden concerns (Carrotte et al., 2025). In addition, in a recent survey of 550 adults with a range of visible differences, 137 (24.9%) reported that podcasts were a preferred format for support delivery (Clement et al., 2025). A podcast series was, therefore, considered a possible mode of delivery for Loving ACTion, and this suggestion was deemed appropriate during PPI (Public and Patient Involvement) input throughout this project, from adults with lived experience of the issues being addressed.

The present study aimed to assess the effectiveness of Loving ACTion, using a mixed methods survey with data collection at three time points (baseline, post‐intervention, 2‐month follow‐up). The primary research question was: ‘Is Loving ACTion associated with reducing appearance‐related distress in romantic and intimate relationships in adults with a visible difference?’ It was hypothesized that:

1. There will be a decrease in appearance‐related distress in romantic and intimate relationships, body image life disengagement, appearance‐fixing behaviours, fear of negative evaluation and internal shame between baseline and post‐intervention, and between baseline and follow‐up.
2. There will be an increase in body esteem, psychological flexibility, valued living and self‐compassion between baseline and post‐intervention, and between baseline and follow‐up.

## METHOD

### Study design

The study employed a concurrent mixed methods approach (Creswell, 2021) to evaluate Loving ACTion, an intervention designed to support adults with a visible difference in relation to intimacy and romantic relationships. Quantitative data were collected using survey methodology through online questionnaires completed at three time points: (1) baseline, (2) post‐intervention and (3) 2‐month follow up. Informed by patient and public involvement (PPI), a pre–post evaluation design was selected for this initial study to maximize access to the intervention, reduce barriers to participation and provide an assessment of change over time.

A pre‐study power calculation, together with pragmatic considerations, was used to determine the target sample size. Because the primary quantitative analyses were planned as two‐sided paired‐samples *t*‐tests (*α* = .05, power = .80), we estimated that approximately 51–66 participants with complete post‐intervention outcome data would be required to detect within‐participant effects in the range d*z* = .40 to .35. Allowing for up to 50% attrition between baseline and post‐intervention, this implied a baseline recruitment target of approximately 100–130 participants. In total, 153 participants completed baseline measures; 76 provided complete baseline–post‐intervention data and 71 provided complete baseline–follow‐up data.

Qualitative data were collected from open‐ended questions in the post‐ and follow‐up surveys. A sub‐set of the sample also took part in one‐to‐one online interviews conducted by the second author, which are reported in a separate publication.

### Participants

Any adult (over 18 years) with an appearance‐affecting condition, injury or treatment effect of any kind was eligible to participate. Participants were required to have a good understanding of spoken and written English as the intervention materials are currently only available in English.

Member organizations of the Appearance Collective (an informal network of more than 30 UK‐based charities that offer support to individuals and families affected by visible difference) were approached for their support with recruitment. These organizations shared details about the research on their social media pages, websites, in newsletters and at events. Research adverts were sent to members of the Centre for Appearance Research participant pool mailing list and posted through its social media pages. These organizations are all UK‐based but their social media followers can be based anywhere, so information about the study did reach some individuals who were outside the United Kingdom. Participants were recruited between February 2024 and April 2025.

A total of 154 adults were recruited at baseline. In this sample, 131 participants identified as female, 21 identified as male, one as non‐binary and one participant preferred to not disclose their gender. The mean age was 42.10 years (range 19–69 years, SD = 13.35). Seven participants identified as Asian/Asian British, 12 as Black/African/Caribbean/Black British, eight as being from mixed or multiple ethnic groups, two as another ethnic group and 125 as White. Twelve participants were currently dating (8%), seven were divorced (5%), 18 were in a relationship and cohabiting (12%), 12 were in a relationship and not cohabiting (8%), 50 were married or in a civil partnership (32%), and 51 were single (33%). In terms of sexuality, participants identified as asexual (*n* = 3, 2%), bisexual (*n* = 8, 5%), demisexual (*n* = 2, 1%), heterosexual (*n* = 127, 82%), lesbian (*n* = 3, 2%), pansexual (*n* = 4, 3%), queer (*n* = 1, 1%) and questioning (*n* = 1, 1%). Participants reported a diverse range of visible differences, including alopecia, birthmarks, burn injuries, craniofacial conditions, facial paralysis, lipoedema, scarring and skin conditions. See Table 1 for a summary of demographic information.

**TABLE 1:** Participant demographic information.

	*N*	%
Gender		
Female	131	85
Male	21	14
Non‐binary	1	1
Prefer not to disclose	1	1
Age		
19–69 (*M* = 42.10, SD = 13.35)	154	
Ethnicity		
Asian/Asian British	7	5
Black/African/Caribbean/Black British	12	8
Mixed/Multiple ethnic groups	8	5
White	125	81
Other ethnic group	2	1
Sexuality		
Asexual	3	2
Bisexual	8	5
Demisexual	2	1
Heterosexual	127	82
Lesbian	3	2
Pansexual	4	3
Queer	1	1
Questioning	1	1
Missing	5	3
Relationship status		
Dating	12	8
Divorced	7	5
In a relationship (cohabiting)	18	12
In a relationship (not cohabiting)	12	8
Married/Civil partnership	50	32
Single	51	33
Other	3	2
Missing	1	1
Country		
Australia	2	1
Canada	3	2
India	1	1
Ireland	7	5
Israel	1	1
Norway	1	1
United Kingdom	128	83
United States	11	7
Visible difference^a^		
Alopecia	21	13
Birthmark	11	7
Burn injury	20	13
Colostomy/Ileostomy	3	2
Craniofacial condition	11	7
Ectodermal dysplasia	1	1
Facial paralysis	19	12
Kyphosis	1	1
Limb difference	2	1
Lipodystrophy	5	3
Lipoedema	31	20
Lumpectomy	1	1
Lymphoedema	8	5
Neurofibromatosis	1	1
Scarring	15	10
Skin condition	14	9
Thyroid eye disease	3	2

^a^ Participants could report more than one visible difference.

### Patient and public involvement (PPI)

Two adults with lived experience of a visible difference and who had expressed an interest in becoming involved with this area of research provided feedback on the study design and materials. This was conducted through online one‐to‐one meetings and email communication where individuals reviewed materials and provided feedback, which was discussed and implemented by the research team.

### Materials

#### Intervention materials

Loving ACTion is a self‐guided evidence‐based intervention aimed at reducing appearance‐related distress in romantic and intimate relationships for adults with a visible difference. The content of this intervention was developed based on existing research into the experiences of romantic relationships, intimacy and disclosure (Sharratt et al., 2018; Sharratt, 2020). The intervention materials are presented in a series of seven audio podcast episodes (duration ranging from 13 to 47 min) and based on the therapeutic model of Acceptance and Commitment Therapy (ACT). The podcast episodes are presented by researchers and Experts by Experience, using a script based on extensive interviews with people with visible differences who have had concerns around these issues. As a group, the podcast hosts vary in terms of gender, sexuality, age, ethnicity, relationship status and experience and type of visible difference. Loving ACTion is focussed on communicating the core concepts of ACT: (1) cognitive defusion (ability to detach not ‘fuse’ with thoughts, images and memories); (2) experiential acceptance; (3) contact with the present moment (ability to consciously and flexibly pay attention to what is happening in the present); (4) values (chosen desired qualities or psychological action); (5) committed action (behaviour in accordance with an individual's values); and (6) self‐as‐context (ability to observe cognitions, emotions and behaviour) (Harris, 2019).

The intervention provides educational content about these concepts and introduces individuals to skills that can help them to manage difficult thoughts and feelings. Alongside communicating the core concepts of ACT, Loving ACTion addresses several topics related to developing and maintaining intimate and romantic relationships. This includes appearance anxiety, communication and disclosure within relationships and sex and physical intimacy. It includes a mixture of content facilitated by the research team and conversations with individuals with lived experience of a visible difference. A range of activities including freely available YouTube videos and downloadable PDF worksheets based on core ACT concepts (e.g., values clarification, mindful breathing, unhooking from thoughts, self‐compassionate letter writing) are embedded within the podcast episodes.

Loving ACTion was developed utilizing a rigorous and iterative co‐production approach. Firstly, a Delphi consensus exercise (*n* = 20) was conducted with psychosocial specialists (self‐reported as being clinical psychologists, counsellors and therapists) and researchers, each with more than 3 years' professional experience of visible difference and/or romantic relationships. These professionals completed a series of surveys to identify and prioritize targets for intervention (e.g., cognitions, affect and emotional difficulties, behaviours, etc.). The findings of the Delphi study indicated that ACT was the most appropriate therapeutic approach to adopt and suggested the intervention content should focus on internal shame, appearance‐related distress and avoidant behaviours. Public involvement user representatives (adults with lived experience of a visible difference, *n* = 6) then each took part in two one‐to‐one collaborative discussions to inform the development of the intervention content and format. Following this, 22 adults with a range of visible differences reviewed a pilot version of Loving ACTion and gave feedback via an online survey collecting both qualitative and quantitative data. Both the content and format of the intervention were found to be acceptable. Many reported that Loving ACTion addressed an unmet psychosocial support need and provided an opportunity to learn skills to manage their own thoughts, feelings and concerns related to intimacy and romantic relationships.

Psychosocial outcome measures were chosen that directly assess constructs key to ACT and the content of the Loving ACTion intervention, specifically, appearance‐related distress in relationships.

#### Appearance‐related distress in relationships

Centre for Appearance Research Romantic Relationships and Intimacy Scale (CARRIS‐17, Sharratt, 2020) is a 17‐item scale that asks respondents to consider a range of items related to romantic relationships and intimacy. Items are scored on a 6‐point scale ranging from 1 (‘strongly disagree’) to 6 (‘strongly agree’). This scale has been specifically developed for use with adults with a visible difference and was the primary outcome measure in this study. It demonstrated good internal consistency at baseline (*α* = .81), post‐intervention (*α* = .82) and follow‐up (*α* = .86).

#### Body image life disengagement

The Body Image Life Disengagement Questionnaire (Atkinson & Diedrichs, 2021) is a 9‐item scale used to measure the degree of participants' disengagement from appearance‐salient activities across different life domains (e.g., social, occupational, etc.). Items are scored on a 4‐point scale ranging from 1 (‘has not stopped me at all’) to 4 (‘stopped me all the time’). This scale has demonstrated good internal consistency at baseline (*α* = .86), post‐intervention (*α* = .87) and follow‐up (*α* = .89).

#### Body image coping strategies

Body Image Coping Strategies Inventory‐Appearance Fixing subscale (BICSI‐AF; Cash et al., 2005) is a 10‐item subscale which is used to assess participants' appearance‐fixing behaviours, such as concealment of appearance difference, altering appearance or seeking reassurance from others. Items are scored on a 4‐point scale ranging from 0 (‘definitely not like me’) to 3 (‘definitely like me’). This scale demonstrated good internal consistency at baseline (*α* = .78), post‐intervention (*α* = .81) and follow‐up (*α* = .85).

#### Body esteem

The Body Esteem for Adolescents and Adults, Appearance subscale (BESAA‐A; Mendelson et al., 2001) is a 10‐item subscale which aims to measure participants' evaluation of their own appearance. Items are rated on a 5‐point scale ranging from 0 (‘never’) to 4 (‘always’). This scale has demonstrated high internal consistency across baseline (*α* = .92), post‐intervention (*α* = .92) and follow‐up (*α* = .93).

#### Fear of negative appearance evaluation

The Fear of Negative Appearance Evaluation Scale (FNAES; Lundgren et al., 2004) is a 6‐item scale which is used to assess participants' concerns that others may negatively evaluate their appearance. Items are scored on a 5‐point scale ranging from 1 (‘not at all’) to 5 (‘extremely’). It has demonstrated high internal consistency across baseline (*α* = .89), post‐intervention (*α* = .91) and follow‐up (*α* = .93).

#### Internal shame

The External and Internal Shame Scale (EISS; Ferreira et al., 2020) is an 8‐item scale used to assess self‐focussed negative evaluations and feelings about the self, associated with external and internal shame. The internal shame sub‐scale is comprised of 4 items scored on a 5‐point scale ranging from 0 (‘never’) to 4 (‘always’). It demonstrated good internal consistency at baseline (*α* = .78), post‐intervention (*α* = .78) and follow‐up (*α* = .81).

#### Psychological flexibility

The Comprehensive Assessment of Acceptance and Commitment Therapy Processes (CompACT; Francis et al., 2016) is a 23‐item scale assessing psychological flexibility across three sub‐scales: (1) openness to experience, (2) behavioural awareness and (3) valued action. Items are scored on a 7‐point scale, ranging from 0 (‘strongly disagree’) to 6 (‘strongly agree’). Both the full CompACT scale (baseline *α* = .86, post‐intervention *α* = .89 and follow‐up *α* = .91) and the openness to experience (baseline *α* = .74, post‐intervention *α* = .78 and follow‐up *α* = .85), behavioural awareness (baseline *α* = .82, post‐intervention *α* = .86 and follow‐up *α* = .89) and valued action (baseline *α* = .81, post‐intervention *α* = .87 and follow‐up *α* = .86) subscales demonstrated good internal consistency across all time points.

#### Valued living

Valued Living Questionnaire (VLQ; Wilson et al., 2010) is a 20‐item scale that asks respondents to rate 10 different life domains (e.g., family, parenting, marriage), indicating their level of importance and how consistently they have lived in accordance with these values in the last week. Items are scored on a 10‐point scale ranging from 1 (‘not at all important/not consistent with my value’) to 10 (‘extremely important/completely consistent with my value’). Two items were included from this scale, one which asked participants to reflect on ‘Marriage/couples/intimate relationships’ and one that asked them to reflect on ‘Friends/social life’.

#### Self‐compassion

The Self‐Compassion Scale Short‐Form (SCS‐SF; Raes et al., 2011) is a 12‐item scale with items scored from 1 (‘almost never’) to 5 (‘almost always’). This scale showed good internal consistency at baseline (*α* = .87), post‐intervention (*α* = .88) and follow‐up (*α* = .89).

#### User engagement measures

Participants were asked to provide self‐report data on which episodes of Loving ACTion they listened to and whether they practiced any of the embedded exercises.

#### Open‐ended questions

The baseline survey asked participants what their visible difference was, with a free‐text response. Open‐ended questions were also included at the end of the post‐intervention and follow‐up surveys. These questions read ‘Do you have anything else that you'd like to share?’ These questions were included to enable participants to elaborate on their experiences and provide context or greater depth to their answers (O'Cathain & Thomas, 2004).

### Procedure

This study was awarded ethical approval from the University of the West of England, College of Health, Science and Society Research Ethics Committee (ref no. HAS.23.05.110). Eligible adults with a visible difference were provided with a detailed information sheet that outlined the content and format of the ‘Loving ACTion’ intervention and the procedure for taking part in the evaluation. Participants were required to provide informed consent using an online form, hosted on the Qualtrics survey platform. They completed baseline measures hosted on the same survey platform and were then emailed a private link to the first podcast episode of the ‘Loving ACTion’ series. Following this, participants were emailed a link to two episodes of the ‘Loving ACTion’ series each week over the next 3 weeks. These emails also included any support materials relevant to those particular episodes, such as links to YouTube videos and worksheets attached as PDF documents. In addition to email reminders, participants could also opt‐in to receive text messages to prompt them to listen to the podcast episodes.

Four weeks after receiving the first podcast episode, participants were emailed a link to the post‐intervention outcome measures to complete and asked if they would like to opt‐in to a nested qualitative study involving one‐to‐one interviews about their experience of using ‘Loving ACTion’. These individual online MS Teams interviews are reported elsewhere.

**FIGURE 1:**
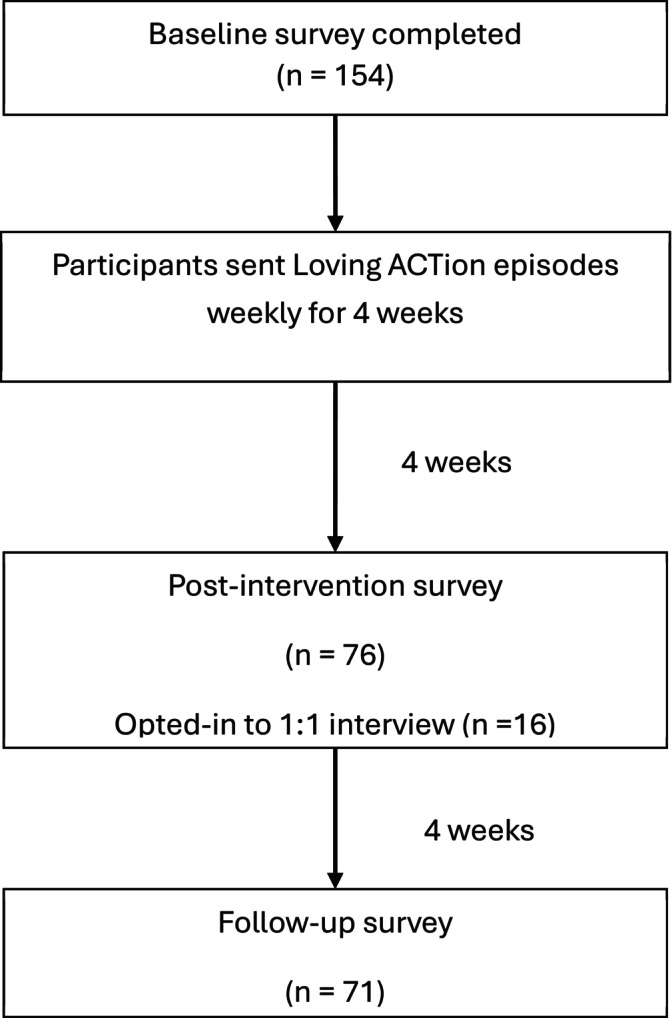
Participant flow through the study.

Finally, 8 weeks (2 months) after completion of the baseline survey, participants were sent a link to the follow‐up outcome measures. At post‐intervention and follow‐up data collection time points, participants were emailed a shopping voucher as a thank you for their time. Figure 1 summarizes participant flow through the study, which was pre‐registered on the Open Science Framework.

### Data analysis

Paired sample *t*‐tests were used to assess changes in mean response between baseline and post intervention, and between baseline and follow‐up since these were the two practically meaningful contrasts specified for this initial evaluation. In light of attrition across timepoints, we chose paired analyses in order to maximize use of available data for each comparison rather than a one‐way repeated‐measures ANOVA, which would have restricted the analysis to complete cases at all three assessments.

The standardized effect size for a repeated measures design was used to estimate Cohen's *d*, using formula 10 in Lakens (2013) where absolute values of *d* may be broadly understood as .01 < *d* < .02 indicating a very small effect, .2 < *d* < .5 as a small effect, .5 < *d* < .8 as a medium effect, .8 < *d* < 1.2 as a large effect and *d* > 1.2 as a very large effect (see Sawilowsky, 2009).

A missing values analysis was undertaken. Participants not providing any data post‐intervention were considered as drop‐outs. Those who dropped out after baseline were compared to those providing some data post‐intervention on categorical demographic variables using the chi‐squared test of association and on their baseline data using the independent sample *t*‐tests. No significant differences were found between those who remained in the study and those who dropped out at baseline. Multiple imputed chained equations (MICE) were used to impute missing data (100 imputations), and sensitivity analyses were performed for reporting as Supporting Information. User engagement data are presented descriptively. All analyses were undertaken using SPSS version 30. Two‐sided tests were conducted throughout with a nominal significance level of *α* = .05.

An exploratory mediation analysis using linear regression analyses examined whether change in psychological flexibility (CompACT) was associated with change in appearance‐related distress in relationships (CARRIS‐17) over time (rather than treating baseline CARRIS‐17 as causing follow‐up CARRIS‐17 via a mediator).

### Open‐ended responses

Open‐ended responses were analysed using content analysis. The fourth author (AZ) extracted data from the online survey platform Qualtrics, transferred it to a Microsoft Excel spreadsheet, anonymized any identifying comments and conducted initial coding. These codes were then refined through their additional readings of the data. They developed initial categories, which were then reviewed in discussion with the first author (MT). These authors collectively generated and reviewed categories and sub‐categories before finalizing them in discussion with the remaining authors.

## RESULTS

Missing values analysis indicated that those consenting to the study but not providing post‐intervention outcome data did not significantly differ from those retained, either on demographic characteristics or on baseline psychosocial measures. As such there was no evidence of systematic differences between completers and non‐completers. For this reason, we report statistical comparisons based on available case data and additionally provide the corresponding comparison on the multiple imputed data set in the Supporting Information, noting that the same broad conclusions are obtained under both analysis sets.

Table 2 summarizes sample size, mean and standard deviation for outcome measures at baseline, post‐intervention and follow‐up. Table 3 summarizes the paired sample *t*‐test analyses and estimated effect size. A summary of means, standard mean error and paired *t*‐test analyses for imputed data is given in the Supporting Information (Table S1).

**TABLE 2:** Means and standard deviations for outcome variables.

Measure	Baseline			Post‐intervention			Follow‐up		
	*N*	Mean	SD	*N*	Mean	SD	*N*	Mean	SD
CARRIS‐17	76	65.03	14.84	76	60.18	13.91	71	57.25	15.24
Body image life disengagement	76	1.94	.69	76	1.78	.67	71	1.66	.66
Body image coping strategies	76	1.75	.56	76	1.72	.56	71	1.71	.59
Body esteem	76	13.20	8.29	76	14.46	7.91	71	15.42	8.21
Fear of negative appearance evaluation	76	20.79	6.02	76	18.99	5.88	71	17.75	6.23
Internal shame	76	8.97	3.79	76	8.04	3.58	71	7.65	3.65
Psychological flexibility	76	70.84	15.99	76	73.68	16.43	71	77.27	17.81
Valued living composite	76	50.93	23.22	76	49.80	21.25	71	55.99	22.25
Self‐compassion	76	2.63	.74	76	2.76	.75	71	2.87	.76
Psychological flexibility subscales									
Openness to experience	76	26.78	8.41	76	27.51	8.25	71	29.76	9.24
Behavioural awareness	76	14.71	5.41	76	15.83	5.04	71	16.38	5.65
Valued action	76	29.36	6.50	76	30.34	6.89	71	31.13	6.91

**TABLE 3:** Summary of paired *t*‐tests comparing baseline to post‐intervention and baseline to follow‐up.

Measure	Baseline mean (SD)	Post‐intervention mean (SD)	*t*	df	*p*	*D*
Baseline to post‐intervention						
CARRIS‐17	65.03 (14.84)	60.18 (13.91)	4.28	75	<.001	.34
Body image life disengagement	1.94 (.69)	1.78 (.67)	2.89	75	.005	.24
Body image coping strategies	1.75 (.56)	1.72 (.56)	.48	75	.635	.09
Body esteem	13.20 (8.29)	14.46 (7.91)	−2.45	75	.017	.16
Fear of negative appearance evaluation	20.79 (6.02)	18.98 (5.88)	3.29	75	.002	.30
Internal shame	8.97 (3.79)	8.04 (3.58)	3.09	75	.003	.25
Psychological flexibility	70.84 (15.99)	73.68 (16.43)	−2.13	75	.037	.18
Valued living composite	50.93 (23.22)	49.80 (21.25)	.50	75	.622	.05
Self‐compassion	2.63 (.74)	2.76 (.75)	−2.05	75	.044	.17
Psychological flexibility subscales						
Openness to experience	26.39 (8.39)	29.76 (9.24)	−3.73	70	<.001	.40
Behavioural awareness	14.56 (5.37)	16.38 (5.65)	−3.43	70	<.001	.33
Valued action	29.07 (6.50)	31.13 (6.91)	−3.00	70	.004	.31

Outcome	Baseline mean (SD)	Follow‐up mean (SD)	*t*	df	*p*	*D*
Baseline to Follow‐up						
CARRIS‐17	65.03 (15.22)	57.25 (15.24)	6.17	70	<.001	.51
Body image life disengagement	1.93 (.70)	1.66 (.66)	4.31	70	<.001	.40
Body image coping strategies inventory	1.76 (.54)	1.71 (.59)	.78	70	.439	.09
Body esteem	12.86 (8.14)	15.42 (8.21)	−3.87	70	<.001	.31
Fear of negative appearance evaluation	20.85 (6.04)	17.75 (6.23)	5.10	70	<.001	.51
Internal shame	9.10 (3.77)	7.65 (3.65)	3.65	70	<.001	.39
Psychological flexibility	70.03 (15.75)	77.27 (17.81)	−4.27	70	<.001	.43
Valued living composite	50.52 (23.12)	55.99 (22.25)	−2.25	70	.028	.24
Self‐compassion	2.58 (.73)	2.87 (.76)	−3.85	70	<.001	.39
Psychological flexibility subscales						
Openness to experience	26.39 (8.39)	29.76 (9.24)	−3.73	70	<.001	.04
Behavioural awareness	14.56 (5.37)	16.38 (5.65)	−3.43	70	<.001	.33
Valued action	29.07 (6.50)	31.13 (6.91)	−3.00	70	.004	.31

### Baseline and post‐intervention

Paired sample *t*‐tests indicated that between baseline and post‐intervention there was a significant decrease in appearance concerns related to intimacy and relationships *t*(75) = 4.28, *p* = <.001, *d* = .34; body image life disengagement *t*(75) = 2.89, *p* = .005, *d* = .24; fear of negative appearance evaluation *t*(75) = 3.29, *p* = .002, *d* = .30; and internal shame *t*(75) = 3.09, *p* = .003, *d* = .25. There was a statistically significant increase in body esteem *t*(75) = 2.45, *p* = .017, *d* = .16; psychological flexibility *t*(75) = 2.13, *p* = .037, *d* = .18; and self‐compassion *t*(75) = 7.13, *p* = <.001, *d* = .17.

### Baseline and follow‐up

Paired sample *t*‐tests indicated that between baseline and follow‐up, there was a significant decrease in appearance concerns related to intimacy and relationships *t*(70) = 6.17, *p* < .001, *d* = .51; body image life disengagement *t*(70) = 4.31, *p* < .001, *d* = .40; fear of negative appearance evaluation *t*(70) = 5.10, *p* < .001, *d* = .51; and internal shame *t*(70) = 3.65, *p* < .001, *d* = .39. There was a statistically significant increase in body esteem *t*(70) = 3.87, *p* < .001, *d* = .31; psychological flexibility *t*(70) = 4.27, *p* < .001, *d* = .43; valued living *t*(70) = 2.25, *p* = .028, *d* = .24; and self‐compassion *t*(70) = 3.65, *p* < .001, *d* = .39.

### Association between psychological flexibility and outcomes

The association between change in psychological flexibility (CompACT) and change in anxiety related to romantic relationships (CARRIS‐17) was assessed using linear regression. Change in CompACT was significantly associated with a change in CARRIS‐17, with a one‐point increase in the CompACT change score associated with a 0.3‐point decrease in the CARRIS‐17 change score (beta = .30, *p* < .001). The model accounted for 15.5% of the variance in CARRIS‐17 change score. This indicates a moderate relationship between improvements in CompACT and reductions in CARRIS‐17 over the study period.

In exploratory analyses, greater improvement in overall psychological flexibility (CompACT total score) over the study period was significantly associated with improvement in body image life disengagement (BILD; *p* = .007), body image coping strategies (BICSI‐AF; *p* = .004), body esteem (BESAA‐A; *p* < .001) and internal shame (*p* < .001).

Exploratory multivariable linear regression analyses using the changes in CompACT subscales (openness to experience, behavioural awareness, valued action) suggested that not all domains of psychological flexibility were associated with outcome change after adjustment for the other CompACT subscales. Changes in openness to experience were significantly associated with changes in appearance‐related distress in relationships (CARRIS‐17; *p* = .008), body image coping strategies (BICSI‐AF; *p* = .048), body esteem (BESAA‐A; *p* = .006), fear of negative appearance evaluation (FNAE; *p* = .039) and internal shame (*p* = .008). Changes in behavioural awareness were significantly associated with changes in appearance‐related distress in relationships (CARRIS‐17; *p* = .003), body image coping strategies (BICSI‐AF; *p* = .008) and body esteem (BESAA‐A; *p* = .003). Changes in valued living were significantly associated only with changes in fear of negative appearance evaluation (FNAE; *p* = .011). These exploratory findings suggest that changes in openness and awareness may have been more closely related to improvements in psychosocial outcomes than changes in valued action, although these analyses should be interpreted with caution.

### User engagement

Participants reported which episodes of the podcast series they had listened to. In total, 72% of participants listened to all seven podcast episodes. Ninety‐two percent (*n* = 70) reported that they listened to the introductory episode and episode two, which begins to discuss the experiences and possible challenges related to having a visible difference, as well as introducing ACT concepts. Most participants (89%, *n* = 68) also listened to episode three, which addresses thoughts and feelings, and episode four, which is focussed on self‐compassion. Fewer respondents then listened to episode five (valued action, 86%, *n* = 66), episode six (being present, 81%, *n* = 62) and episode seven (communication in relationships, 76%, *n* = 58). Fifty‐eight (76%) participants reported having tried at least one of the activities embedded within the podcast episodes.

Exploratory analyses were conducted to examine whether engagement with the intervention was associated with change in outcomes over the study period. Participants were grouped into higher‐ and lower‐engagement categories and compared on outcome change scores. These analyses did not indicate a consistent association between engagement level and improvement in outcomes. The only nominally significant between‐group difference was for FNAE (*p* = .049), but this was in the opposite direction to that expected under a simple dosage‐effect expectation, with greater improvement observed in the lower‐engagement group. This is an isolated finding and should be interpreted cautiously given the exploratory nature of the analysis and the number of outcomes examined.

At post‐intervention, 68 participants also responded to a question about their preferred format for the Loving ACTion content. Forty participants (59%) reported that they felt the current format (an audio podcast series) was most appropriate. Several other formats were suggested including an e‐book (*n* = 38, 56%), a physical booklet (*n* = 29, 43%) or a smaller summary document (*n* = 25, 37%).

### Open‐ended responses

Three categories were generated from the content analysis of the open‐ended survey responses. The first category, ‘A real difference’, highlighted participants' positive experiences with the podcast episodes. They shared that they found the content educational, relatable, and that it had made a real difference to their lives. These positive differences included examples of where exercises had helped them to navigate difficult thoughts about their visible difference. Participants also emphasized the benefits of hearing other people's personal stories and how this had made them feel less isolated when managing difficult situations.

The second category, ‘Every person's journey is different’, captured participants' awareness that everyone who listens to the podcast will be coming from different places in their experiences with romantic relationships and intimacy. Participants acknowledged that the content may be more psychologically challenging for some than others.

The third category, ‘Future development’, focussed on some aspects of the podcast that participants felt were less effective or engaging (e.g., some meditation exercises) and suggestions for developments (e.g., adding easier navigation and playback speed options). A few participants (*n* = 4) also reported that the content felt too scripted and this reduced their engagement with the intervention. See Table 4 for a summary of the categories and example quotes.

**TABLE 4:** Summary of content analysis categories with example quotes.

Category	Frequency of codes	Description	Illustrative quotes
A real difference	87	Participants highlighted the positive aspects of the experience that they enjoyed while completing the podcast. They also shared how this experience has made a real difference to their life and had changed the way they approach their thought processes about themselves by using analogies introduced in the episodes.	‘I had never considered the way that I am is a bus driver. It has very much helped me in processing mentally the way I think and feel with regards to my thoughts regarding my legs. The way they look, the way that they feel. Those passengers on the bus who simply won't shut up!!’ ‘It is helpful to revisit the podcasts and reinforce the acceptance they bring. I think that's what helps to be kinder to oneself’. ‘Thank you for sharing other experiences in the interviews I could relate and made me feel less alone’
Every person's journey is different	7	Participants shared how individual insecurities and coping levels will impact the experience of the podcast. They also highlighted that visible differences are wide‐ranging and exist on a spectrum, so people will have very different experiences with the podcast depending on their experiences with visible difference.	‘Every day is different and some days its easier to cope and deal with than others. I wonder if an acquired difference plays out differently to a lifelong condition’. ‘I feel my injuries are worse because they are all over my head and face and so cannot be disguised’. ‘I found session 6 very difficult to complete due to my own insecurities and therefore am yet to complete the practices from sessions 6 and 7’.
Future development	12	Participants suggested that some aspects of the podcast could be improved in the future. These included changing or removing some exercises, making the content feel less scripted, and developing the characters introduced in the episodes.	‘Found the talks stilted. They were reading from a sheet ABOUT how to mindful or accepting etc rather than BEING IN THE STATE of being mindful. Talking about it rather than being in it makes a difference and it felt very low impact’. ‘I got quite lost and a bit frustrated with the ABC table in ep. 5. It felt very rushed and I didn't want to spend time on it’. ‘The meditations in the podcasts are kind of annoying. There are lots of other resources online for guided breathing type exercises. This is such a unique opportunity to reach a specific subgroup of people, it feels kind of like a waste of time to shoehorn those in’.

Overall, the quantitative findings indicate that Loving ACTion was associated with meaningful improvements across several domains, and that these changes were maintained at follow‐up, suggesting that participants continued to apply the skills introduced in the intervention. Importantly, the qualitative data provide additional insight into how and why these changes may have occurred. Taken together, the convergence of quantitative and qualitative findings strengthens confidence in the overall pattern of results. The qualitative accounts offer contextual detail that aligns closely with the observed statistical improvements, suggesting that the ACT‐based components of Loving ACTion were experienced as both relevant and applicable in participants' daily lives. This integration of findings provides a more comprehensive understanding of this novel intervention's impact and highlights the potential value of ACT‐informed approaches for adults with visible differences navigating romantic and intimate relationships.

## DISCUSSION

This study evaluated Loving ACTion, a self‐guided ACT‐based intervention designed to support adults with visible differences in relation to romantic relationships and intimacy. Participation in the intervention was associated with reductions in appearance concerns related to romantic relationships, body image life disengagement, fear of negative evaluation and internal shame, as well as increases in body esteem, psychological flexibility and self‐compassion. These patterns were maintained at follow‐up with additional improvements observed in valued living. Changes in appearance‐related distress in relationships were also moderately associated with changes in psychological flexibility, which suggests a relationship between these outcomes over time.

Significant improvements in psychosocial outcomes indicate that Loving ACTion was helpful in supporting adults to both manage appearance‐related distress and develop skills to increase psychological flexibility. Responses to open‐ended questions indicated that participants found the podcast engaging, valuable and felt that it made a real difference in their lives. Alongside this, a number of participants provided recommendations for future developments to the intervention. These findings align with evaluations of ACT‐based interventions for adults with visible differences. For example, a study with adults with facial palsy reported significant improvements in psychological well‐being, social function and appearance‐related distress after completing an information and therapy guide that included ACT‐based content (Hotton et al., 2022). Similarly, a mobile app intervention for adults with visible differences of any kind has indicated promising feasibility and initial positive findings, with users reporting reduced body image life disengagement and improvements in psychological flexibility (Zucchelli et al., 2022). In addition, an early ACT‐based intervention aimed at supporting burn injured adults with appearance concerns was found to be acceptable to patients and could support reductions in appearance‐related distress (Shepherd et al., 2026). However, none of the above interventions focus specifically on concerns related to romantic relationships and intimacy. Alongside support from existing literature, the findings of the present study provide preliminary evidence supporting the potential value of Loving ACTion as a novel intervention for adults with a visible difference and that ACT‐based interventions may have an important role to play in future support development for this population.

While not statistically significant, improvements in the appearance fixing subscale of the coping strategies index (BICSI‐AF) were reported and were associated with improvements in key ACT constructs measured in this study, namely psychological flexibility, openness to experience and behavioural awareness. This tentatively suggests that the ACT‐based intervention could reduce users' reliance on strategies such as camouflaging and disguising in attempts to reduce distress associated with their visible difference. However, this warrants further investigation.

Within the open‐ended responses, participants highlighted the value of having lived experience voices as part of the intervention content and that hearing other people's stories had made them feel less alone. This emphasizes the positive impact of peer support that may benefit adults with visible differences. For example, a qualitative study with 12 adults with alopecia found that users of online support groups reported that these spaces helped them to express their feelings, seek practical advice and make connections with others (Iliffe & Thompson, 2019). However, while another qualitative study with adults with alopecia (Davey et al., 2019) also found that engagement with peer support had mostly been positive, some participants also reported that contact with others with alopecia had been distressing because it reminded them that a full recovery was unlikely. It is, therefore, important to balance potential benefits of peer support with concerns related to possible distress. The scripted podcast format meant that content was moderated and extensive PPI input throughout the development of the podcast episodes helped us to ensure that the shared lived experiences of people with visible differences who have had concerns about relationships were portrayed in a sensitive and appropriate manner, in order to reduce the chance of users feeling upset by the content and/or language.

Self‐report user engagement indicated that most participants (72%) listened to all seven episodes in the podcast series. This indicates a high level of engagement and acceptability of the content and mode of delivery of the intervention. A recent scoping review of podcast‐based interventions for improving mental health outcomes (e.g., body image, mindfulness and stigma reduction) has indicated that there is increasing evidence to support the efficacy of this mode of delivery (Carrotte et al., 2025). Additionally, previous research exploring the impact of an early intervention podcast for individuals with eating disorders found that the privacy and autonomy associated with listening to a podcast was important for users and enhanced the efficacy of the intervention (Tatham et al., 2026). The topics covered by Loving ACTion (i.e., appearance concerns, sex and relationships) may be sensitive or emotive to users and so the accessibility of the podcast format may have contributed to the observed effects in the present study. Consequently, podcast‐based interventions could be a promising and accessible format for future psychosocial interventions focussed on appearance concerns.

### Implications

The present research suggests that an ACT‐based therapeutic approach may be acceptable to adults with visible differences and may be associated with psychological outcomes relevant to intimacy and romantic relationships. This may have important clinical relevance for identifying key targets for intervention to support psychological wellbeing in adults with visible differences. Psychological interventions that focus on teaching skills to foster psychological flexibility and self‐compassion may help reduce appearance concerns and improve well‐being. Loving ACTion is now available as a free resource that provides clinicians and support professionals with accessible and evidence‐based materials to share with service users. This novel resource meets a current gap in the provision of psychological support by addressing a sensitive and often challenging topic for adults with a visible difference.

### Limitations

The sample in this study was self‐selecting and so may not be representative of all adults with visible differences who require support related to intimacy and romantic relationships. As noted in the open‐ended responses, individuals have varying experiences and needs, and some may not be psychologically ready to explore this topic. Users of the intervention are advised at the outset that it is not suitable for those experiencing severe distress, who feel suicidal or are self‐harming, or want individual support from a healthcare professional.

Future research could usefully explore whether participant characteristics moderated intervention effects. This was not possible with the current sample. Most participants (*n* = 131, 85%) were women and heterosexual (*n* = 127, 82%). Although common within psychological research, this may mean that it is unclear whether the intervention is effective for individuals of all gender identities and sexualities. Existing research suggests appearance norms do vary between genders and sexualities (Hayfield, 2013) and not conforming to these appearance norms due to having a visible difference can be distressing and impact intimacy and romantic relationships for LGBTQIA+ people (Waite, 2025). Future research should aim to be more inclusive of LGBTQIA+ adults to better understand their experiences and support needs.

Additionally, the majority of participants identified as White (*n* = 125, 81%). The research exploring experiences of individuals with visible differences in non‐Western cultures is limited. However, there have been cultural differences noted in the general population in terms of appearance norms (Landor et al., 2024) and the role of physical appearance in relationships (Anderson et al., 2008). Additionally, differences in experiences have been noted within the visible difference literature. For example, young Somali adults with facial differences have described experiences shaped by negative beliefs about visible difference within the Somali community (Costa, 2022). As a result, it may not be possible to generalize findings of the present study to adults from other cultural backgrounds. Loving ACTion is currently only recorded in English, which may have excluded some individuals from participating and will act as a barrier to broader accessibility. Future research should consider translating this intervention into different languages to facilitate wider implementation. It would also be useful to know more about the contexts in which people engaged with the podcast series and whether (and if so, how) this was used alongside other potential sources of support.

Finally, the study design did not involve randomization nor a control or comparison group. Given the scarcity of support relating to romantic relationships and intimacy currently available specifically for this group, these methodological decisions were made to prioritize increasing access to this support and were informed by discussions with PPI representatives. A pre–post design was chosen to maximize ecological validity and reduce barriers to participation, but this approach limits the internal validity of the findings. Without a control group, we cannot rule out alternative explanations for the observed improvements over time, such as regression to the mean, spontaneous improvement over time, or the influence of additional factors or external life events. As such, causal inferences regarding the effectiveness of Loving ACTion should be made cautiously. Future research could benefit from employing a randomized controlled or quasi‐experimental design and a longitudinal approach to more rigorously test the intervention's efficacy, better isolate the specific contribution of the ACT‐based content, and determine the longer‐term impact of the intervention.

## CONCLUSION

The present study aimed to assess the effectiveness of Loving ACTion, a novel ACT‐based intervention for adults with visible differences focussed on concerns related to romantic relationships and intimacy. Between baseline and post‐intervention, statistically significant reductions were found in appearance‐related distress in romantic relationships, body image life disengagement, fear of negative appearance evaluations and internal shame, and significant improvements were found in body esteem, self‐compassion and psychological flexibility. Additionally, between baseline and follow‐up, the same associations were found plus statistically significant improvements in valued living. Open‐ended responses indicated that Loving ACTion made a real difference to participants' lives and was a valuable resource. These findings suggest that this is an acceptable and effective novel intervention for adults with visible differences. Loving ACTion could be an important resource for health professionals and support organizations to help address a gap in support related to intimacy and romantic relationships. Future research could explore the effectiveness of the intervention for a broader range of genders and sexualities and consider translating Loving ACTion into languages other than English to increase accessibility. Loving ACTion can be accessed for free at www.VisibleDifferenceSupportHub.com.

## AUTHOR CONTRIBUTIONS

**Maia Thornton:** Conceptualization; investigation; writing – original draft; methodology; formal analysis; project administration; data curation. **Emma Waite:** Writing – review and editing; formal analysis; investigation. **Paul White:** Writing – review and editing; formal analysis; methodology. **Anna Zarola:** Investigation; writing – review and editing. **Yara Vizinho:** Investigation; writing – review and editing. **Alex Clarke:** Supervision; methodology; writing – review and editing. **Diana Harcourt:** Funding acquisition; writing – review and editing; supervision; resources; methodology.

## FUNDING INFORMATION

This work was supported by the VTCT Foundation.

## CONFLICT OF INTEREST STATEMENT

The authors declare that they have no known competing financial interests or personal relationships that could have appeared to influence the work reported in this paper.

## Supporting information


Table S1.

## Data Availability

Due to ethical restrictions, data cannot be shared. Data include outcomes and demographic information provided by adults with visible differences (some of which are rare conditions) and sharing the data may result in identification of these individuals. The University of the West of England College of Health, Science and Society Research Ethics Committee has not given approval to share the raw data, and consent has also not been provided by individual participants.
