# Supplementary material for: Loving ACTion: An evaluation of an ACT‐based audio podcast intervention focussed on romantic and intimate relationships for adults with visible differences
Source: Br J Health Psychol. 2026 Jul 28;31(3):e70096. doi: 10.1111/bjhp.70096 (PMC13411655; doi:10.1111/bjhp.70096)
Supplement: Supplementary file 1 — Table S1. [file BJHP-31-0-s001.docx]

Table S1: Supplementary material. Summary of paired t-test analyses using imputed data.

| **Baseline to Post-intervention** | | | | |
| --- | --- | --- | --- | --- |
| Outcome | Mean  Difference | Standard error of the mean difference | t | *p* |
| CARRIS | 4.84 | 1.13 | 4.28 | <.001 |
| Body image life disengagement | 1.58 | .055 | 2.89 | .004 |
| Body image coping strategies | 0.03 | .055 | 0.477 | .633 |
| Body esteem | -1.26 | 0.52 | -2.45 | .014 |
| Fear of negative appearance evaluation | 1.80 | 0.55 | 3.29 | .001 |
| Internal shame | 0.93 | 0.30 | 3.09 | .002 |
| Psychological flexibility | -2.84 | 1.33 | -2.13 | .033 |
| Valued living composite | 1.14 | 2.30 | .495 | .621 |
| Self-compassion | -0.13 | 0.66 | -2.05 | .041 |
| **Psych flex subscales** |  |  |  |  |
| Openness to experience | -0.74 | 0.71 | -1.04 | .300 |
| Behavioural awareness | -1.12 | 0.47 | -2.32 | .017 |
| Valued action | -0.99 | 0.62 | -1.60 | .110 |
| **Baseline to Follow-up** | | | | |
| Outcome | Mean  Difference | Standard error of the mean difference | t | *p* |
| CARRIS | 7.84 | 1.30 | 6.05 | <.001 |
| Body image life disengagement | 0.26 | 0.06 | 4.12 | <.001 |
| Body image coping strategies | 0.06 | 0.06 | 1.07 | .286 |
| Body esteem | -2.50 | 0.68 | -3.39 | <.001 |
| Fear of negative appearance evaluation | 3.10 | 0.61 | 5.04 | <.001 |
| Internal shame | 1.37 | 0.39 | 3.48 | <.001 |
| Psychological flexibility | -6.64 | 1.72 | -3.87 | <.001 |
| Valued living composite | -5.06 | 2.55 | -1.98 | .047 |
| Self-compassion | -0.29 | 0.73 | -3.88 | <.001 |
| **Psych flex subscales** |  |  |  |  |
| Openness to experience | -3.04 | 0.92 | -3.30 | <.001 |
| Behavioural awareness | -1.76 | 0.56 | -3.15 | .002 |
| Valued action | -1.91 | 0.71 | -2.68 | .007 |
